# Case report: Two cases of leptomeningeal metastases in patients with metastatic urothelial carcinoma treated with enfortumab vedotin

**DOI:** 10.3389/fonc.2024.1434814

**Published:** 2024-11-29

**Authors:** Francine Fishbein, Lucia Nappi, Behnoush Mortazavi, Bernhard Eigl

**Affiliations:** ^1^ Department of Medicine, University of British Columbia, Vancouver, BC, Canada; ^2^ Department of Medicine, Medical Oncology Division, British Columbia (BC) Cancer, Vancouver Centre, University of British Columbia, Vancouver, BC, Canada; ^3^ Department of Medicine, Medical Imaging Division, British Columbia (BC) Cancer, Vancouver Centre, University of British Columbia, Vancouver, BC, Canada

**Keywords:** leptomeningeal carcinomatosis, urothelial carcinoma, enfortumab vedotin, case report, metastatic urothelial carcinoma

## Abstract

**Background:**

Leptomeningeal carcinomatosis is an exceptionally rare pattern of metastases in genitourinary cancer, described in less than 0.1% of cases. We report two cases of patients with metastatic urothelial cancer who initially responded to enfortumab vedotin (EV) before developing leptomeningeal metastases.

**Case presentation:**

Case 1: A 55 year-old man was diagnosed with metastatic urothelial carcinoma. He was initially treated with cisplatin/gemcitabine chemotherapy, followed by second-line pembrolizumab, with progression on both of these regimens. He was started on EV therapy and had a sustained partial response. After 12 cycles of treatment, he developed neurologic symptoms with imaging showing extensive leptomeningeal metastases. A lumbar puncture was performed with cytology positive for metastatic carcinoma. Case 2: A 63 year-old man was diagnosed with metastatic urothelial carcinoma. He received 6 cycles of platinum/gemcitabine chemotherapy followed by avelumab maintenance, after which he developed radiographic progression. He was started on EV therapy and developed a complete radiographic response. After 13 cycles of treatment, he developed neurologic symptoms and imaging revealed extensive leptomeningeal disease. Cytology confirmed metastatic urothelial carcinoma.

**Conclusion:**

This uncommon pattern of spread observed in two patients treated with EV in short succession represents a potentially significant and novel pattern of progression within this population.

## Introduction

Leptomeningeal carcinomatosis refers to metastatic spread of cancer to the arachnoid and pia mater (1). It is an exceedingly uncommon complication of genitourinary cancers, reported in only 0.03% of cases (2). A recent literature review described only 33 known cases of leptomeningeal carcinomatosis in bladder cancer to date (3). Since this report, there have been a few additional described cases (4–6), however it remains a very rare entity within bladder cancer patients. Here, we report a case series of 2 patients with metastatic urothelial carcinoma on enfortumab vedotin (EV) who subsequently developed leptomeningeal metastases. EV is a novel and effective treatment for patients with metastatic urothelial cancer, consisting of an antibody-drug conjugate directed against Nectin-4, a protein highly expressed in urothelial carcinoma cells (7). EV has been shown to prolong survival in patients with advanced urothelial cancer previously treated with platinum-based chemotherapy and a PD-1/PD-L1 inhibitor, and more recently, the combination of EV and pembrolizumab has demonstrated superiority over platinum-based chemotherapy in the first-line metastatic setting (8, 9). While EV has shown promising results in these trials, its efficacy within the central nervous system (CNS) and patterns of progression remain poorly understood. Patients with active CNS metastases were excluded from these trials. This report of 2 cases of leptomeningeal metastases in patients treated with EV highlights an important, novel pattern of CNS spread within this population.

## Case description: case 1

**Figure 1:**
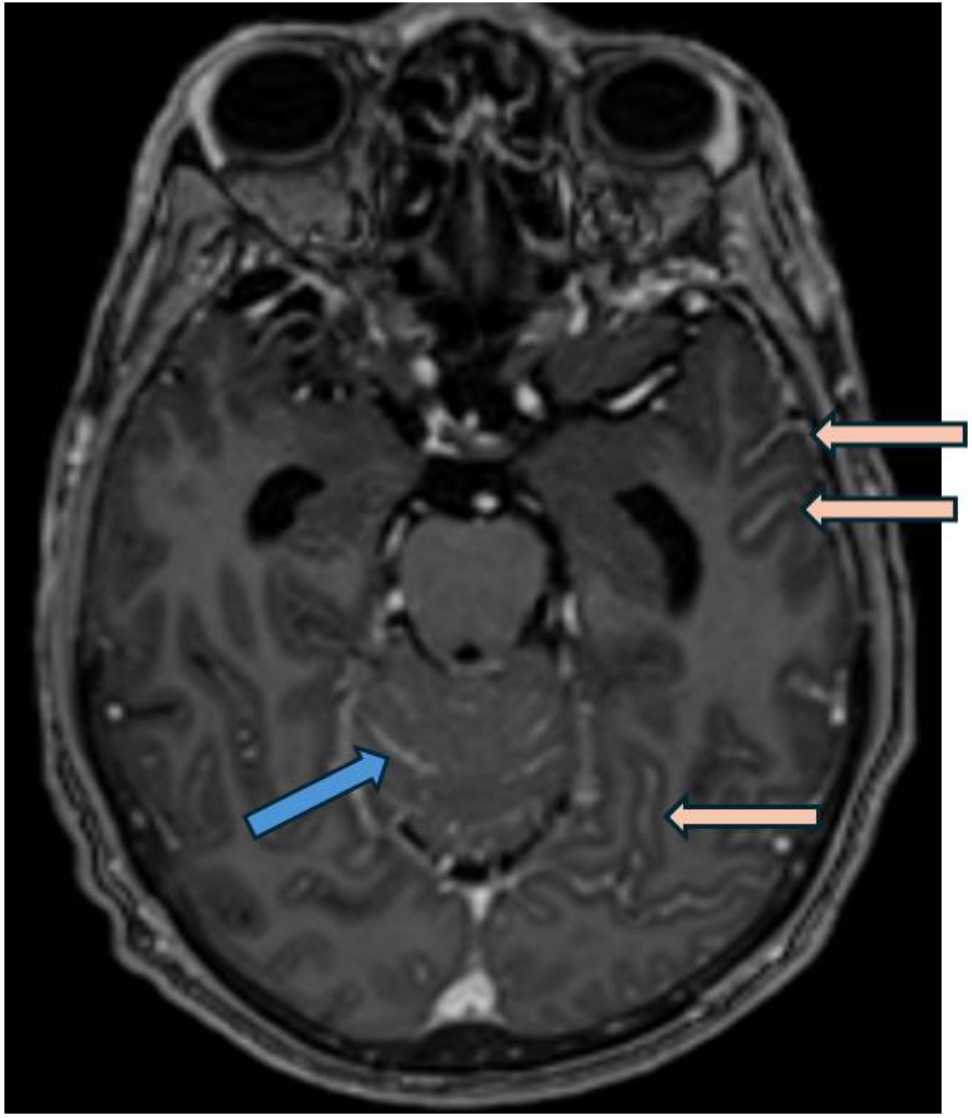
Post gadolinium axial T1 weighted MRI (MP-RAGE, i.e.,: Magnetization Prepared - RApid Gradient Echo) demonstrates smooth leptomeningeal thickening and enhancement along the vermis and cerebellar folia in posterior cranial fossa (blue arrow) and along the temporo-occipital sulci in the cerebrum (pink arrows), consistent with leptomeningeal carcinomatosis.

**Figure 2:**
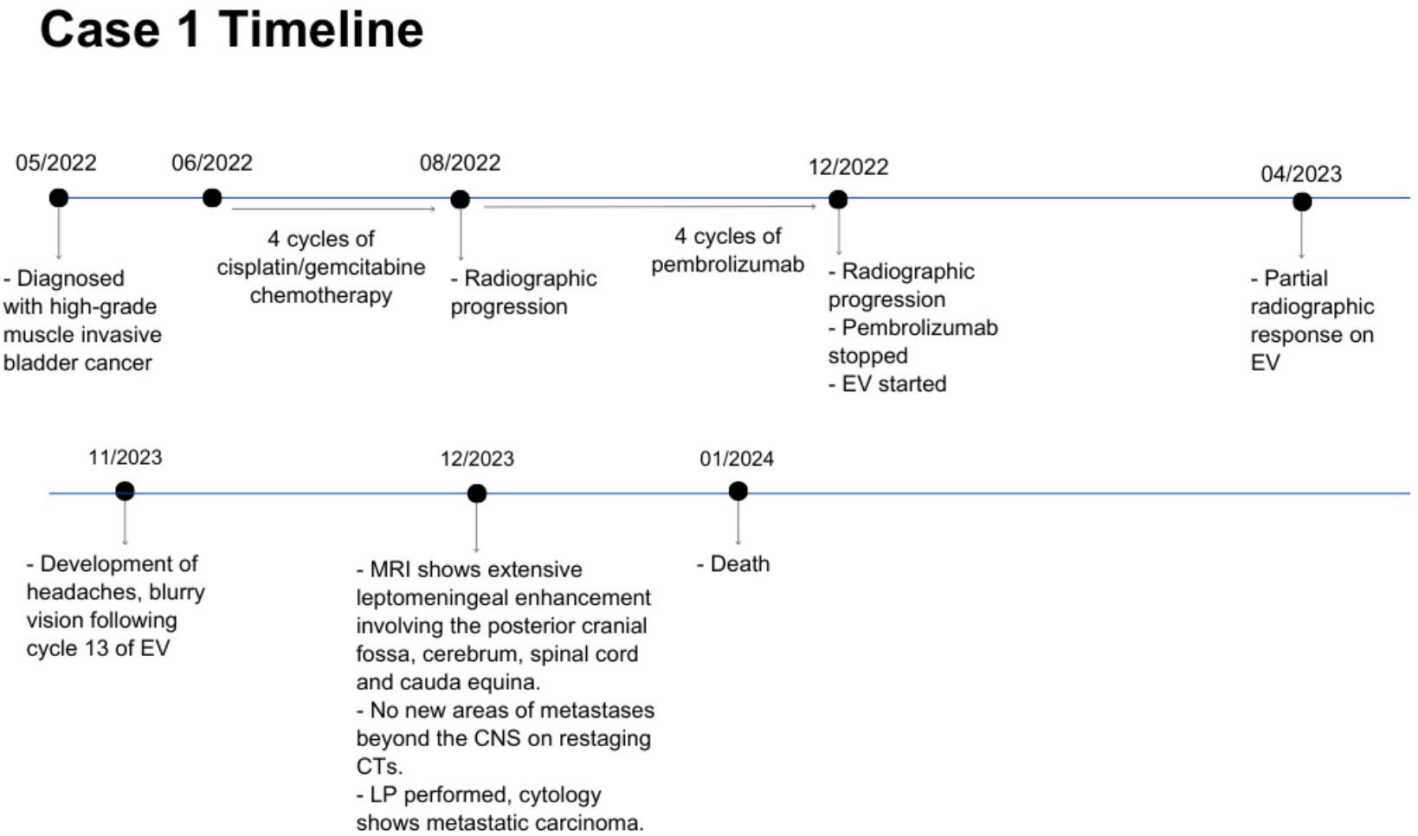
Case presentation of patient 1. EV, enfortumab vedotin; CTs, computed tomography scans; CNS, central nervous system; LP, lumbar puncture.

A 55 year-old man was diagnosed with metastatic high-grade muscle invasive bladder cancer in 2022. He initially presented with hematuria and underwent a transurethral bladder tumor resection revealing a muscle invasive urothelial carcinoma, as well as an area of high-grade urothelial carcinoma with focal anaplastic features. Staging investigations revealed para-aortic, iliac, pelvic sidewall and retroperitoneal lymphadenopathy. He completed 4 cycles of cisplatin/gemcitabine chemotherapy, with subsequent radiographic progression. He had ongoing progression on second-line pembrolizumab with worsening lymphadenopathy. He was then started on EV in December 2022, at which time his lymph nodes were the only sites of metastases. His best response on EV was a partial response, with significant improvement in his lymphadenopathy which was sustained throughout treatment. In November 2023, following cycle 12 of treatment (11 months total), he began to develop headaches, blurry vision, and balance issues. A CT angiogram showed prominent leptomeningeal enhancement in the cerebellum and bilateral cerebral hemispheres, suspicious for leptomeningeal metastasis. An MRI demonstrated extensive leptomeningeal enhancement within the posterior cranial fossa and the cerebrum, the cord and cauda equina (**Figure 1**). A lumbar puncture confirmed cytology consistent with metastatic carcinoma. Restaging CT scans showed interval enlargement of his bladder mass, with no other areas of new metastases. He was seen by Radiation Oncology in consultation, who felt that craniospinal radiation would be unlikely to provide symptom or mortality benefit in his case. He was managed supportively and passed away in hospital shortly thereafter (**Figure 2**).

## Case description: case 2

**Figure 3:**
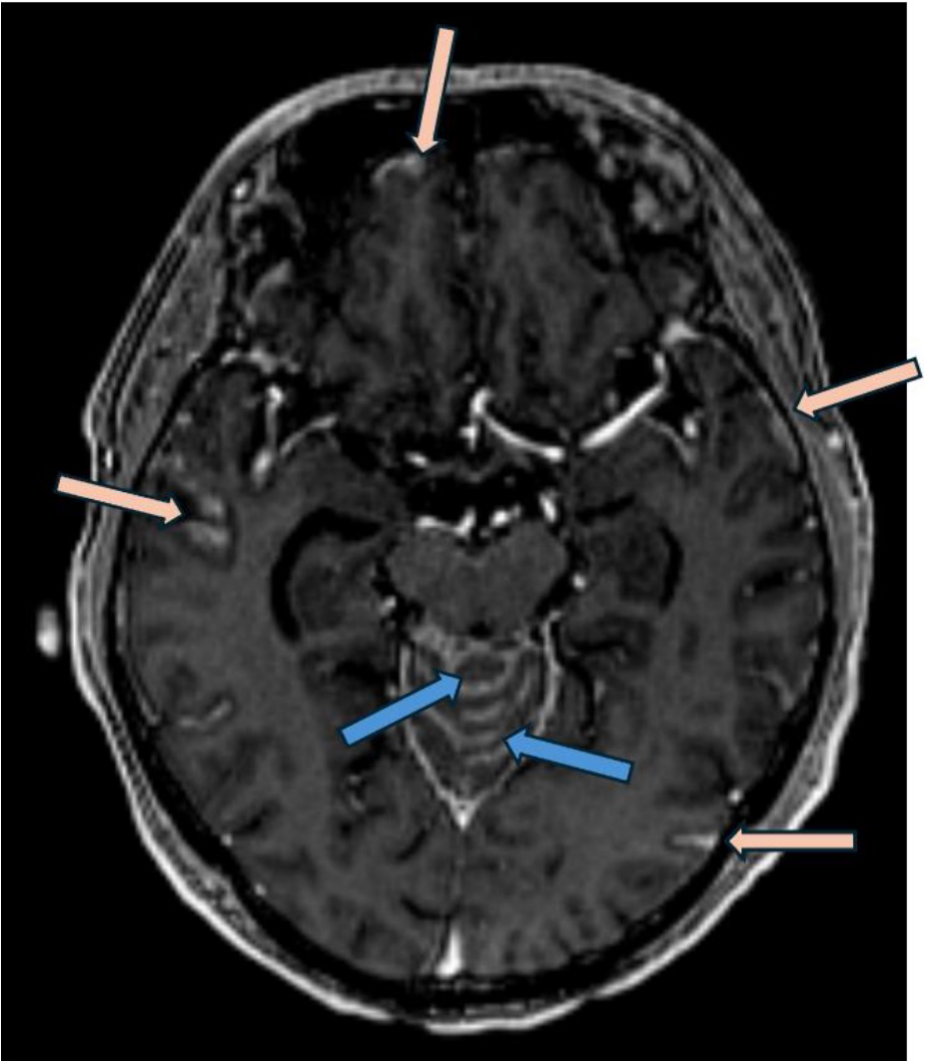
Post gadolinium axial T1 weighted MRI (MP-RAGE, ie: Magnetization Prepared - RApid Gradient Echo) demonstrates nodular leptomeningeal enhancement involving the vermis of the cerebellum (blue arrows) in addition to temporal and frontal lobes (pink arrows).

**Figure 4:**
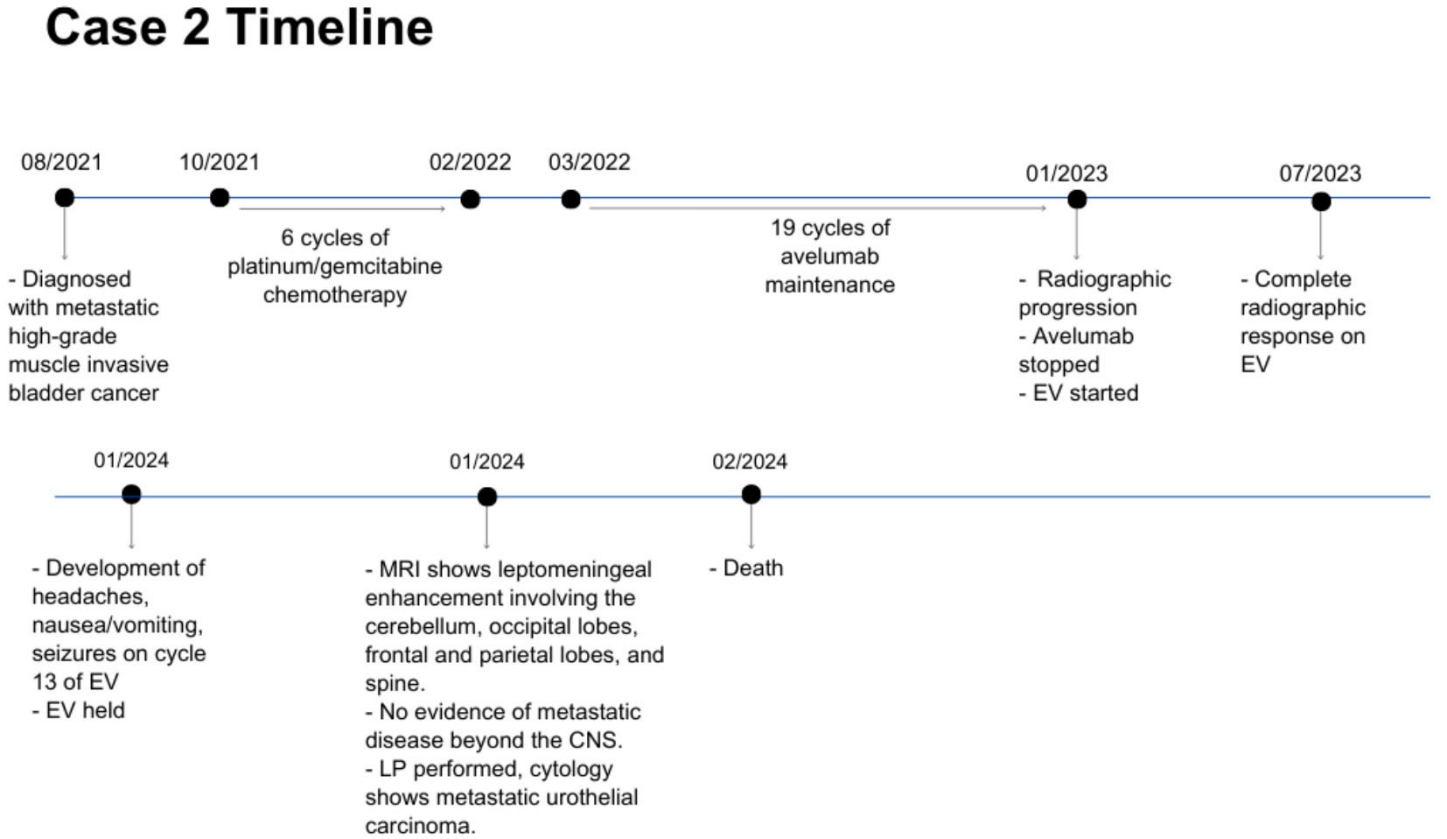
Case presentation of patient 2. EV, enfortumab vedotin; CNS, central nervous system; LP, lumbar puncture.

A 63 year-old man was diagnosed with metastatic high-grade muscle invasive bladder cancer in 2021. He presented with hematuria and underwent a transurethral bladder tumor resection revealing invasive high-grade urothelial carcinoma. Staging investigations revealed periaortic, iliac and retroperitoneal lymphadenopathy. He received 6 cycles of platinum/gemcitabine chemotherapy followed by avelumab maintenance for 19 cycles. Upon radiographic progression, he started on EV therapy in January 2023, at which point the lymph nodes were the only sites of metastases. He had a complete response to EV, with no measurable disease seen on subsequent CT scans. In January 2024, while on cycle 13 of EV therapy (12 months total), he began to develop headaches, nausea and vomiting, and seizures. An MRI revealed leptomeningeal enhancement involving the cerebellum, occipital lobes, frontal and parietal lobes, and spine (**Figure 3**). There was no evidence of metastatic disease beyond the CNS. A lumbar puncture was performed, with cytology consistent with metastatic urothelial carcinoma. His case was discussed with Radiation Oncology, however it was not felt that he would benefit from or be able to tolerate craniospinal radiation. He was managed supportively, and passed away shortly after (**Figure 4**).

## Discussion

Leptomeningeal carcinomatosis is extremely rare in bladder cancer, described in just over 30 patients with bladder cancer to date (3). Here, we describe 2 recent cases of leptomeningeal carcinomatosis in patients with metastatic urothelial carcinoma on EV therapy. This highlights a potentially important pattern of spread within this population. The underlying mechanism for this spread is not yet understood. One possibility is that EV has poor CNS penetration, and the meninges represent a sanctuary site. Enfortumab vedotin has not been well-studied within the CNS, with only 2 previous case series describing patients with CNS disease on EV (5, 6). One case series described 3 patients with CNS disease who responded to EV, while the other described 9 patients with CNS progression during or after treatment with EV. Interestingly, each series had 1 patient with leptomeningeal disease - one of whom responded to EV, and one who developed leptomeningeal disease while on EV. It is therefore not yet clear whether EV is effective in treating leptomeningeal disease.

Another possibility is that there is a subset of bladder cancer patients, not yet described, who have a propensity to develop leptomeningeal metastases. In non-small cell lung cancer, for example, it has been described that patients with EGFR mutations who have been treated with tyrosine kinase inhibitor (TKI) therapy are more likely to develop leptomeningeal disease (10, 11). One explanation for this is that these patients develop mutations conferring resistance to TKI therapy, with disease relapse manifesting as leptomeningeal metastases within the CNS. The acquired T790M mutation is one such known mutation, conferring first and second generation-TKI resistance. In patients with this mutation, osimertinib (a third-generation TKI), has been shown to improve clinical outcomes, including in patients who develop leptomeningeal disease (12, 13). There may be a similar phenotype in bladder cancer that we are only beginning to see now, as survival for these patients increases with novel treatments such as EV. Interestingly, the 2 patients we describe both had metastatic disease limited to the lymph nodes at the time of EV initiation, with initial excellent response to EV before the development of leptomeningeal disease. Recognizing this phenotype would have important clinical ramifications, representing an opportunity for targeted therapy in the future. Currently, the treatment for leptomeningeal carcinomatosis in bladder cancer patients is largely supportive, and prognosis remains poor (4). An identified molecular target within these patients has the possibility to significantly improve outcomes in this population.

Several potential mechanisms of resistance to EV exist. Decreased expression of Nectin-4 in patients with metastatic urothelial carcinoma has been shown to be associated with EV resistance (14). Resistance to the payload through upregulation of the P-glycoprotein drug efflux pump has also been described in *in vitro* and *in vivo* models (15, 16). Other proposed mechanisms include mutations affecting antigen binding, internalization and trafficking, and the cell cycle (17). There are similarly several described mechanisms of CNS tropism leading to the development of brain metastases. These include: an underlying genetic predisposition for detachment, dissemination and blood brain-barrier penetration; metabolic adaptation through transcriptomic and epigenetic changes in metastatic colonies; the development of an inflammatory microenvironment in the brain; and immune evasion preventing T-cell mediated cancer cell destruction (18). Likely, a combination of these factors resulted in our patients’ development of leptomeningeal progression, however a deeper understanding of these mechanisms in patients with metastatic urothelial carcinoma treated with EV is essential.

Finally, our report brings up the need for further research on the indications for craniospinal irradiation (CSI) in the management of leptomeningeal carcinomatosis. Currently, the use of CSI for management of leptomeningeal disease relies upon expert opinion, typically indicated in patients with good performance status and with disease outside of the CNS that is stable or for which effective treatment options exist (19). As survival for patients with metastatic urothelial carcinoma improves with effective systemic therapies, these indications may need to be re-evaluated in the future.

Overall, the relationship between EV and leptomeningeal disease remains unclear, but will become increasingly significant as EV moves into the first-line setting in patients with metastatic bladder cancer. While this pattern of spread is exceedingly rare in bladder cancer, this series of two cases in close temporal and geographic proximity who were responding systemically to enfortumab vedotin when they developed leptomeningeal disease highlights a potentially significant pattern of progression that requires further evaluation.

## Data Availability

The original contributions presented in the study are included in the article/supplementary material. Further inquiries can be directed to the corresponding author.

## References

[1] Grossman SA Krabak MJ. Leptomeningeal carcinomatosis. Cancer Treat Rev. (1999) 25:103–19. doi: 10.1053/ctrv.1999.0119 10395835

[2] Yust-Katz S Mathis S Groves MD. Leptomeningeal metastases from genitourinary cancer: the University of Texas MD Anderson Cancer Center experience. Med Oncol. (2013) 30:429. doi: 10.1007/s12032-012-0429-z 23292836

[3] Umezawa Y Shirotake S Kaneko G Nishimoto K Okada Y Uchino A. Meningeal carcinomatosis from bladder cancer: A case report and review of the literature. Mol Clin Oncol. (2019) 10:506–10. doi: 10.3892/mco.2019.1820 PMC6466998 31007911

[4] Tomioka M Kawase M Kato D Takai M Iinuma K Horie K. Leptomeningeal carcinomatosis in urothelial carcinoma of the urinary bladder: A report of a patient with a fulminant course who died of cancer after definitive therapies. Case Rep Urol. (2021) 2021:5543939. doi: 10.1155/2021/5543939 34012689 PMC8105107

[5] Shipp C Jindal T Chou J Friedlander TW Koshkin VS Kumar V. Central nervous system disease progression among patients with metastatic urothelial carcinoma treated with enfortumab vedotin: A case series. Clin Genitourin Cancer. (2023) 22:315–21. doi: 10.1016/j.clgc.2023.11.014 PMC12172690 38114390

[6] Vulsteke C De Cocker L Gómez de Liaño A Montesdeoca C De Meulenaere A Croes L. First evidence of activity of enfortumab vedotin on brain metastases in urothelial cancer patients. Pharm (Basel). (2023) 16:375. doi: 10.3390/ph16030375 PMC10057070 36986475

[7] Maiorano BA Catalano M Maiello E Roviello G. Enfortumab vedotin in metastatic urothelial carcinoma: the solution EVentually? Front Oncol. (2023) 13:1254906. doi: 10.3389/fonc.2023.1254906 37781180 PMC10535083

[8] Powles T Rosenberg JE Sonpavde GP Loriot Y Durán I Lee J-L. Enfortumab vedotin in previously treated advanced urothelial carcinoma. N Engl J Med. (2021) 384:1125–35. doi: 10.1056/NEJMoa2035807 PMC8450892 33577729

[9] Powles T Valderrama BP Gupta S Bedke J Kikuchi E Hoffman-Censits J. Enfortumab vedotin and pembrolizumab in untreated advanced urothelial cancer. N Engl J Med. (2024) 390:875–88. doi: 10.1056/NEJMoa2312117 38446675

[10] Li Y-S Jiang B-Y Yang J-J Tu H-Y Zhou Q Guo W-B. Leptomeningeal metastases in patients with NSCLC with EGFR mutations. J Thorac Oncol. (2016) 11:1962–9. doi: 10.1016/j.jtho.2016.06.029 27539328

[11] Li N Bian Z Cong M Liu Y. Survival outcomes of patients with epidermal growth factor receptor mutations in non-small cell lung cancer with leptomeningeal metastasis. Front Oncol. (2021) 11:723562. doi: 10.3389/fonc.2021.723562 35127465 PMC8811957

[12] Mok TS Wu Y-L Ahn M-J Garassino MC Kim HR Ramalingam SS. Osimertinib or platinum-pemetrexed in EGFR T790M-positive lung cancer. N Engl J Med. (2017) 376:629–40. doi: 10.1056/NEJMoa1612674 PMC6762027 27959700

[13] McLean LS Faisal W Parakh S Kao SC Lewis CR Chin MT. Standard-dose osimertinib in EGFR-mutated non-small-cell lung adenocarcinoma with leptomeningeal disease. JCO Precis Oncol. (2021) 5:561–8. doi: 10.1200/PO.20.00464 34994604

[14] Klumper N Ralser DJ Ellinger J Roghmann F Albrecht J Below E. Membranous NECTIN-4 expression frequently decreases during metastatic spread of urothelial carcinoma and is associated with enfortumab vedotin resistance. Clin Cancer Res. (2023) 29:1496–505. doi: 10.1158/1078-0432.CCR-22-1764 PMC10102834 36534531

[15] Chang KLR Delavan HM Winebaum J Porten SP Feng FY Chu CE. Mechanisms and strategies to overcome resistance to enfortumab vedotin in bladder cancer. J Clin Oncol. (2024) 42:S28–S29. doi: 10.1200/JCO.2024.42.4_suppl.690

[16] Cabaud O Berger L Crompot E Adélaide J Finetti P Garnier S. Overcoming resistance to anti-nectin-4 antibody-drug conjugate. Mol Cancer Ther. (2022) 21:1227–35. doi: 10.1158/1535-7163.MCT-22-0013 35534238

[17] Khoury R Saleh K Khalife N Saleh M Chahine C Ibrahim R. Mechanisms of resistance to antibody-drug conjugates. Int J Mol Sci. (2023) 24:9674. doi: 10.3390/ijms24119674 37298631 PMC10253543

[18] Yuzhalin AE Yu D. Brain metastasis organotropism. Cold Spring Harb Perspect Med. (2020) 10:a037242. doi: 10.1101/cshperspect.a037242 31548224 PMC7197417

[19] Barbour AB Kotecha R Lazarev S Palmer JD Robinson T Yerramilli D. Radiation therapy in the management of leptomeningeal disease from solid tumors. Adv Radiat Oncol. (2024) 9:101377. doi: 10.1016/j.adro.2023.101377 38405313 PMC10885590

